# Headliners: Molecular Biology: Lead, Paraquat, and Methylmercury Disrupt Neuronal Stem Cells by a Common Mechanism

**Published:** 2007-05

**Authors:** Jerry Phelps

Li Z, Dong T, Proschel C, Noble M. 2007. Chemically diverse toxicants converge on Fyn and c-Cbl to disrupt precursor cell function. PLoS Biol 5(2):e35.

The quest to establish general principles of mechanism for both known and potential toxicants is a central challenge that fuels investigations in toxicological research. Now groundbreaking research from NIEHS grantee Mark Noble and colleagues at the University of Rochester Medical Center demonstrates that low levels of diverse environmental agents—namely lead, paraquat, and methylmercury—converge on a previously unrecognized regulatory pathway to disrupt the normal functioning of progenitor cells within the central nervous system.

Previous research indicates that many toxicants can increase cells’ oxidative status, but questions remain as to how this shared ability relates to toxicant function. In the current study, the investigators conducted work in cell cultures of glial progenitors (advanced-stage stem cells important for the growth, development, and normal functioning of the central nervous system). They exposed the cells to sublethal concentrations of lead, paraquat, and methylmercury.

The cells proved exquisitely sensitive to minute levels of the toxicants, and the researchers found a previously unknown mechanism by which these agents cause the effects. They noted that the agents increased cell oxidation, which activated an enzyme known as Fyn kinase. This in turn activated another enzyme known as c-Cbl that modifies and degrades protein receptors necessary for cell division and survival. When the receptors are degraded, their downstream signaling pathways are repressed, and the cells fail to divide and develop properly.

The investigators also dosed six-week-old female SJL mice with methylmercury for 30 to 60 days prior to mating, and then throughout pregnancy and gestation at levels 75–90% lower than concentrations that have been linked to gross defects in adult or developing animals. They observed a subtle but significant reduction in the number of cells engaged in DNA synthesis in animals exposed to 100 ppb methylmercury via maternal drinking water. These results were consistent with observed effects of *in vitro* exposure to low-level methylmercury.

The pathway activated by toxicants such as lead, paraquat, and methylmercury is a normal cellular regulatory pathway; however, according to the authors, these agents “are just activating it inappropriately.” This discovery of a molecular target that is shared by a variety of compounds may represent a new tool for rapidly screening compounds to determine their potential neurotoxicity. It may also provide insights into how to protect cells from signaling disruption once exposure has occurred.

